# Influence of Elasticity of Hydrogel Nanoparticles on Their Tumor Delivery

**DOI:** 10.1002/advs.202202644

**Published:** 2022-08-18

**Authors:** Xiangyu Chen, Shuwei Zhang, Jinming Li, Xiaobin Huang, Haochen Ye, Xuezhi Qiao, Zhenjie Xue, Wensheng Yang, Tie Wang

**Affiliations:** ^1^ State Key Laboratory of Inorganic Synthesis and Preparative Chemistry College of Chemistry Jilin University Changchun 130022 P. R. China; ^2^ Beijing National Laboratory for Molecular Sciences Key Laboratory of Analytical Chemistry for Living Biosystems Institute of Chemistry Chinese Academy of Sciences (CAS) Beijing 100049 P. R. China; ^3^ Department of Orthopedics Chinese PLA General Hospital Beijing 100853 P. R. China; ^4^ Life and Health Intelligent Research Institute Tianjin University of Technology Tianjin 300384 P. R. China

**Keywords:** flow conditions, soft hydrogel nanoparticles, cellular uptake, tumor targeting, cellular adhesion

## Abstract

Polymeric nanocarriers have a broad range of clinical applications in recent years, but an inefficient delivery of polymeric nanocarriers to target tissues has always been a challenge. These results show that tuning the elasticity of hydrogel nanoparticles (HNPs) improves their delivery efficiency to tumors. Herein, a microfluidic system is constructed to evaluate cellular uptake of HNPs of different elasticity under flow conditions. It is found that soft HNPs are more efficiently taken up by cells than hard HNPs under flow conditions, owing to the greater adhesion between soft HNPs and cells. Furthermore, in vivo imaging reveals that soft HNPs have a more efficient tumor delivery than hard HNPs, and the greater targeting potential of soft HNPs is associated with both prolonged blood circulation and a high extent of cellular adhesion.

## Graphical abstract

Tuning the elasticity of polymeric nanocarriers affects their tumor delivery efficiency. The high extent of adhesion between soft nanoparticles and tumor endothelial cells plays an important role in their tumor targeting.

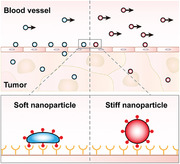

## 1 Introduction

Polymeric nanocarriers have been adopted as a preferred method for drug delivery, as they offer solutions to overcome many problems of the actual therapies.^[^ ^1^ ^]^ Many polymeric nanocarrier‐based drugs have been applied in clinical practice and have made significant contributions to the treatment of diseases such as hepatitis, chronic kidney disease, and oncology.^[^ ^2^ ^]^ However, the inefficient delivery of polymeric nanocarriers to target pathological tissues limits their widespread application, especially in the field of tumor therapy.^[^ ^1a^ ^]^ In previous studies, the tumor delivery efficiency of polymeric nanocarriers has been improved by modifying their size, surface charge, and surface chemistry,^[^ ^3^ ^]^ while mechanical properties are usually overlooked.

Mechanical properties are an important factor in the regulation of biological processes.^[^ ^4^ ^]^ Red blood cells (RBCs) are one of the most common delivery systems in the body and the principal means of delivering oxygen to body tissues. The extraordinary flexibility of RBCs enables them to pass through the blood vessel that has a smaller diameter than themselves and to live in blood circulation for ≈100–120 days. RBCs lose their flexibility as they age and are eventually cleared via reticuloendothelial system (RES).^[^ ^5^ ^]^ Metastatic cancer cells exhibit a lower Young's modulus than healthy cells, and their flexibility is thought to play an important role in their spread to other tissues.^[^ ^6^ ^]^ Therefore, we believe that tuning the elasticity of polymeric nanocarriers will lead to different biological properties and thus affecting their tumor delivery efficiency.

In this study, we prepared hydrogel nanoparticles (HNPs) of different elasticities and modified the tumor‐target molecule cyclic arginyl‐glycyl‐aspartic acid (RGD) on their surface, to study the impacts of mechanical properties and specific interactions of HNPs on tumor delivering. Human umbilical vein endothelial cells (HUVECs) were cultured in a microfluidic system to study their HNP uptake under flow conditions. The results showed that changes in elasticity had a significant impact on cellular uptake of HNPs, and HUVECs showed high uptake potential for soft RGD‐modified HNP (HNP‐RGD) in flow experiments. Animal experiments also showed the influence of modifying elasticity, with soft HNP‐RGD showing superior tumor delivery efficiency. Elasticity control provides a new idea for enhancing the targeting performance of polymeric nanocarriers.

## 2 Results and Discussion

### 2.1 Synthesis and Characterization of HNPs

**Figure 1:**
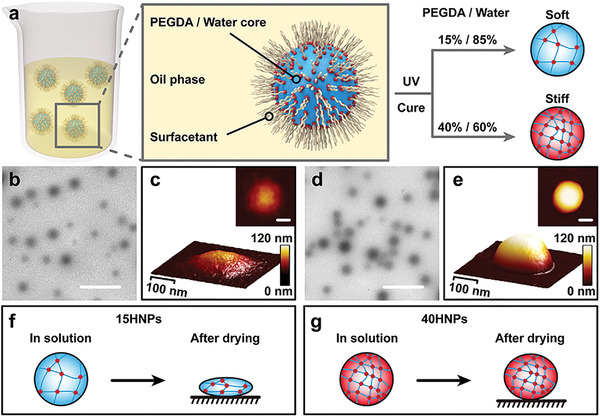
a) Scheme illustrating the synthesis of HNPs. When the PEGDA content was 15% and 40%, the softest and hardest HNPs were obtained, respectively. The softest and hardest HNPs were termed 15HNPs and 40HNPs, respectively. b,d) In situ TEM images of 15HNPs and 40HNPs. c,e) AFM images of 15HNPs and 40HNPs. f,g) The morphologies of 15HNPs and 40HNPs in solution and after drying. Scale bars represent 1 µm for b and d, and 100 nm for c and e.

HNPs were synthesized from poly (ethylene glycol) diacrylate (PEGDA, MW 600), commonly used to synthesize HNPs for biological applications, owing to its good biocompatibility.^[^ ^7^ ^]^ PEGDA and water formed nanoemulsions in oil with the help of surfactants, which was then irradiated with UV light in presence of the photoinitiator 2‐hydroxy‐2‐methylpropiophenone to obtain HNPs (**Figure** **1**a). HNPs with different elasticities were obtained by controlling PEGDA‐to‐water ratio (Figure S1, Table S1, Supporting Information). The softest and hardest HNPs were obtained when the PEGDA contents were 15% and 40%, and they were termed as 15HNPs and 40HNPs, respectively. Mechanical properties of HNPs were characterized using atomic force microscope (AFM) and Young's modulus was measured to be 0.37 MPa for 15HNPs and 3.15 MPa for 40HNPs (Figure S3, Supporting Information). The difference in elasticities of HNPs was mainly because of the difference in their solid contents (Table S1, Supporting Information). Because of the lower solid content of 15HNPs, their aqueous solution was more transparent than that of other HNPs with an equal number of particles (Figure S1, Supporting Information). In situ transmission electron microscopy (TEM) revealed that HNPs were spherical in aqueous solution (Figure 1b,d). AFM analysis showed that 15HNPs collapsed after drying (Figure 1c,f and Figure S5a,b, Supporting Information), while 40HNPs collapsed relatively slightly owing to their higher solid content (Figure 1e,g and Figure S5c,d, Supporting Information). To further investigate the effect of molecular modification on targeting potential, 2‐carboxyethyl acrylate was added to the synthesis process to introduce carboxyl groups on the surface of HNPs, which made them easier to modify. DLS results showed that the hydrodynamic size and zeta potential of HNPs were approximately 310 nm and ‐40 mV, respectively (Table S2, Supporting Information).

### 2.2 Cellular Uptake of HNPs Under Static Conditions

**Figure 2:**
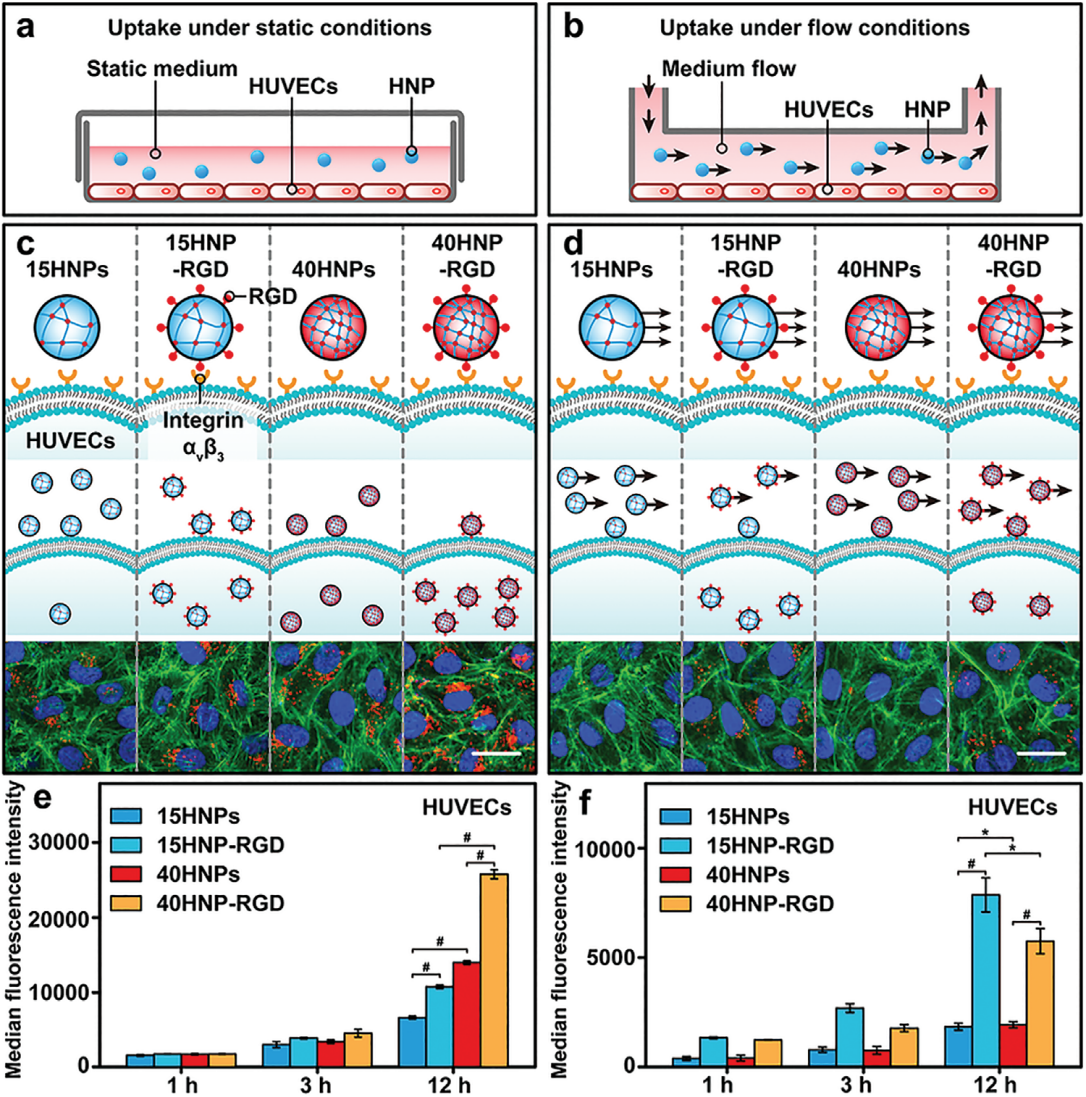
a,b) Scheme illustrating HNP uptake by HUVECs under static and flow conditions. c,d) Fluorescence microscopic images showing uptake of different HNPs by HUVECs under static and flow conditions. Nuclei, cytoskeleton (F‐actin), and HNPs were stained blue, green, and red, respectively. Cellular uptake of 15HNPs, 15HNP‐RGD, 40HNPs, and 40HNP‐RGD under e) static and f) flow conditions, at different time points. All values are the means ± SD (*n* = 3, with *p < 0.05, **p < 0.01, and ^#^ *p* < 0.001; ns, not significant). Scale bars represent 40 µm for c and d.

We first used conventional static conditions to evaluate the effects of mechanical properties and specific interactions on cellular HNP uptake (**Figure** **2**a). To investigate the effect of specific interactions on cellular uptake, we modified RGD on the surfaces of 15HNPs and 40HNPs and named the modified materials 15HNP‐RGD and 40HNP‐RGD, respectively. The successful modification of RGD was verified by Fourier transform infrared spectroscopy examination (Figure S7, Supporting Information). The particle size and zeta potential of HNP‐RGD did not show significant changes after modification (Table S2, Supporting Information). RGD is a common tumor‐targeting molecule and interacts strongly with glioma, melanoma, and endothelial cells with overexpression of integrin *α*_*ν*_ *β*_3_.^[^ ^8^ ^]^ Therefore, HUVECs and human cervical carcinoma cells (HeLa) with integrin *α*_*ν*_ *β*_3_ overexpression, murine macrophage (RAW264.7) and human breast adenocarcinoma cells (MCF‐7) with integrin *α*_*ν*_ *β*_3_ underexpression were used for HNP cellular uptake assays.^[^ ^9^ ^]^ To study the kinetics of cellular uptake, different HNPs were co‐incubated with cells for various time points (1, 3, and 12 h), and the HNPs were loaded with 1,1″‐dioctadecyl‐3,3,3″,3′‐tetramethylindocarbocyanine perchlorate (DiI) dye for quantitative analysis.

NPs are usually quickly cleared by RES in vivo. Avoiding rapid uptake by RES resident macrophages results in longer blood circulation time for NPs.^[^ ^10^ ^]^ The macrophage uptake efficiency of different HNPs can reflect their ability to escape blood clearance. In the present study, changes in mechanical properties of HNPs affected their uptake by RAW264.7 cells, as observed previously.^[^ ^11^ ^]^ The uptake of different HNPs by RAW264.7 cells increased with time, and lower uptake of soft 15HNPs was noticed (Figure S11a, Supporting Information), probably because the deformation of 15HNPs slowed their internalization rate,^[^ ^12^ ^]^ and resulted in a reduced uptake. The results of the macrophage uptake experiments under flow conditions were similar to those under static conditions. Although fluid shear affected the cellular uptake efficiency of HNPs, 15HNPs still exhibited a lower macrophage uptake than 40HNPs (Figure S11b, Supporting Information). RAW264.7 cells have low expression of integrin *α*_*ν*_ *β*_3_; therefore, modification of RGD had a low impact compared to the changes in mechanical properties of HNPs. The low macrophage uptake efficiency of 15HNPs may allow for longer blood circulation compared to 40HNPs. Similar results were obtained for MCF‐7 cells with low integrin *α*_*ν*_ *β*_3_ expression, with only the elasticity of HNPs having a significant influence on cellular uptake (Figure S15, Supporting Information).

As tumor neovascular endothelial cells preferentially express integrin *α*_*ν*_ *β*_3_, HUVECs are widely used as an in vitro model of tumor angiogenic endothelium.^[^ ^13^ ^]^ Recent studies have also found that endothelial cell transport may be the main route through which NPs enter tumors.^[^ ^14^ ^]^ Examination of the *α*_*ν*_ *β*_3_ integrin receptor on HUVECs surface showed that HUVECs displayed a great integrin *α*_*ν*_ *β*_3_ expression (Figure S10, Supporting Information).^[^ ^15^ ^]^ When DiI‐loaded HNPs were co‐incubated with HUVECs for 3 h, substantial uptake of hard 40HNPs was observed in confocal microscopic images (Figure 2c, Figure S13, Supporting Information). Flow cytometry results showed a significantly lower uptake of soft 15HNPs and 15HNP‐RGD than hard 40HNPs and 40HNP‐RGD at 12 h (*p* < 0.001) (Figure 2e), indicating that the elasticity of HNPs greatly affected their uptake by HUVECs. The lower HUVECs uptake of soft 15HNPs was probably because soft NPs have slower internalization rate.^[^ ^16^ ^]^ RGD modification enhanced the cellular uptake, which was significantly higher for HNP‐RGD than for HNPs, by HUVECs (*p* < 0.001), suggesting that specific interactions between HUVECs and HNP‐RGD could promote the uptake. The results were also verified using HeLa cells (Figure S12, Supporting Information). In summary, HUVECs exhibited a high uptake of 40HNPs, and modification of RGD further enhanced the uptake potential.

### 2.3 Cellular Uptake of HNPs Under Flow Conditions

The shearing of blood flow affects the uptake of NPs by cells in vivo.^[^ ^17^ ^]^ Therefore, using a microfluidic system to construct in vitro experiments under flow conditions may help better evaluate the biological properties of NPs. We incubated HUVECs on microfluidic chips and studied the uptake of different HNPs under flow conditions at a shear rate of 300 s^–1^ (Figure 2b, Figure S14, Supporting Information). Confocal imaging showed that the cell membrane remained intact under serial shear of fluid, but HNP uptake significantly reduced compared with that observed under static condition (Figure 2d, Figure S13, Supporting Information). Flow cytometry analysis indicated that the uptake of 15HNPs and 40HNPs by HUVECs at 12 h decreased by 3.7 and 7.8 times, respectively (Figure 2e,f). Uptake of NPs by cells can be divided into two steps: the first step is binding of the NPs to cell surface and the second step is internalization of the NPs.^[^ ^18^ ^]^ Under the static condition, NPs will deposit on the surface of the cell membrane and induce cellular internalization,^[^ ^19^ ^]^ so the uptake efficiency of NPs depends mainly on the rate of internalization. However, the binding of NPs with cell surface is affected by shear force of the fluid under flow conditions. The high shear force will induce huge dislodging forces that are able to detach the adhered NPs.^[^ ^20^ ^]^ Because of the low adhesion of 15HNPs and 40HNPs to cells, they are “washed away” under fluid shear, resulting in low intracellular uptake.

Adhesion between NPs and cells is an important factor that influences cellular uptake under flow conditions.^[^ ^21^ ^]^ Strong interaction between RGD and integrin *α*_*ν*_ *β*_3_ resulted in considerable adhesion of HNP‐RGD to HUVECs^[^ ^15^ ^]^; therefore, the intracellular uptake of 15HNP‐RGD and 40HNP‐RGD was much higher than that of 15HNPs and 40HNPs (Figure 2f). Interestingly, unlike the higher uptake of 40HNP‐RGD under static condition (Figure 2e), the intracellular uptake of 15HNP‐RGD was slightly higher than that of 40HNP‐RGD under flow conditions (*p* < 0.05). The cellular internalization rate of soft NPs is lower than that of hard NPs.^[^ ^16^, ^22^ ^]^ However, the high uptake efficiency of 15HNP‐RGD under flow conditions may be because of its higher adhesion to cells.

### 2.4 Deformable 15HNP‐RGD Exhibits Greater Adhesion than 40HNP‐RGD

**Figure 3:**
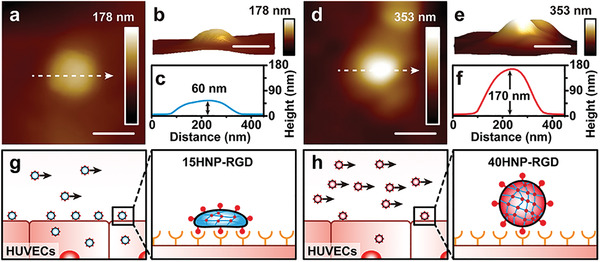
a,d) AFM images of 15HNP‐RGD and 40HNP‐RGD on the surface of HUVECs. b,e) 3D morphologies of 15HNP‐RGD and 40HNP‐RGD. c,f) The cross‐sectional view of the height profile corresponds to the line drawn in a and d. 15HNP‐RGD collapses on the cell surface after binding because of its deformability, which leads to a larger contact area than 40HNP‐RGD. g,h) Scheme illustrator shows that high adhesion between 15HNP‐RGD and HUVECs results in an increased cellular uptake. Scale bars represent 200 nm for a, b, d, and e.

Unlike static conditions, the cellular uptake efficiency of NPs under flow conditions requires consideration of the adhesion of NPs to cell surface.^[^ ^20^ ^]^ Many studies have shown that increasing the contact area between NPs and cells can increase cellular adhesion, and thus increasing the cellular uptake of NPs under flow conditions.^[^ ^21^, ^23^ ^]^ AFM studies showed that 40HNP‐RGD maintained a spherical shape on cell surface (**Figure** **3**d–f). In contrast, 15HNP‐RGD collapsed on cell surface because of its deformability, which led to a larger contact area than that of spherical 40HNP‐RGD (Figure 3a–c). The larger contact area allowed more RGD to interact with integrin *α*_*ν*_ *β*_3_, resulting in greater cell adhesion for 15HNP‐RGD.^[^ ^21^ ^]^ This is similar to the case where the adhesion of cells to the substrate increases with the deformability of cells.^[^ ^24^ ^]^ The greater cell adhesion of 15HNP‐RGD prevents it from being “washed away” by fluid shear, thus showing a higher cellular uptake. Considering that NPs need to adhere to cell surface before being taken up, adhesion potential of NPs may play a significant role in their cellular uptake under flow conditions. This was reflected by a higher cellular uptake of 15HNP‐RGD than that of 40HNP‐RGD under flow conditions, despite a low internalization rate (Figure 3g,h).

### 2.5 In Vivo Study of HNPs

**Figure 4:**
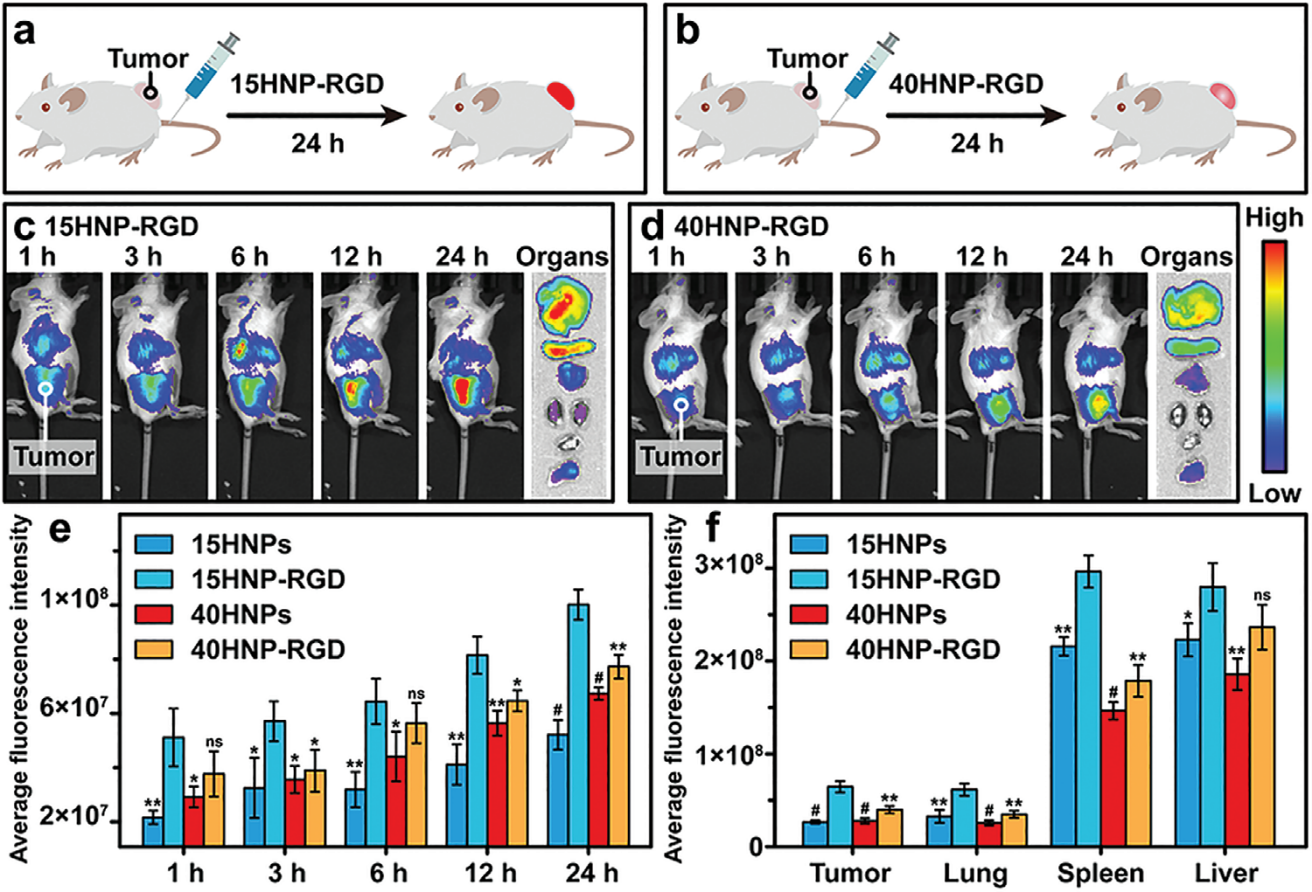
a,b) Scheme illustrating tumor targeting efficiencies of different HNPs. In vivo images of 4T1 tumor xenograft‐bearing mice at different time points, and various organs (liver, spleen, lung, kidneys, heart, and tumor, from top to bottom) collected from mice after 24 h of injecting with c) 15HNP‐RGD and d) 40HNP‐RGD. Average fluorescence intensities of e) tumors at different time points after HNPs injection, and f) different excised mouse organs obtained after 24 h of HNPs injection. Values were compared to those of 15HNP‐RGD. All values are represented as the means ± SD (*n* = 3, with *p < 0.05, **p < 0.01, and ^#^ *p* < 0.001; ns, not significant).

The tumor delivery efficiency of the HNPs in vivo was further investigated using a 4T1 tumor‐bearing BALB/c mouse model, which is a common in vivo tumor angiogenic endothelium model.^[^ ^25^ ^]^ Tumor angiogenic endothelium is commonly used to study the targeting potential of RGD‐modified NPs owing to its preferential expression of *α*_v_ *β*_3_ integrins.^[^ ^26^ ^]^ Following injection with 1,1‐dioctadecyl‐3,3,3,3‐tetramethylindotricarbocyaineiodide (DiR)‐loaded HNPs through the tail vein of mice, the in vivo distribution and tumor‐targeting potential were analyzed at different time points (1, 3, 6, 12, and 24 h) using a small animal imaging system. Results revealed that 15HNP‐RGD efficiently targeted tumors, and showed a high enrichment at the tumor site within a short time period (**Figure** **4**a,e,f). Tumor accumulation of NPs is generally believed to be associated with blood circulation,^[^ ^27^ ^]^ but the longer blood circulation (Figure S16, Supporting Information) and lower tumor enrichment (Figure 4e,f) of 15HNPs do not seem to fully support this view. Therefore, the high extent of adhesion between 15HNP‐RGD and tumor endothelial cells may play an important role in tumor targeting. The high tumor enrichment potential of 15HNP‐RGD can be attributed to the following two aspects.

The first is the deformability of soft HNP‐RGD resulted in greater cellular adhesion. The modification of targeted ligands can increase the interaction of nanoparticles to tumor tissue and thus improving delivery efficiency.^[^ ^28^ ^]^ Compared with hard 40HNP‐RGD, soft 15HNP‐RGD has a larger contact area with tumor endothelial cells owing to its deformability, therefore it leads to stronger adhesion to tumor endothelial cells to further enhance its tumor‐targeting potential. The enrichment of NPs in tumors when increasing the contact area with tumor endothelial cells has also been demonstrated in other studies, where it was found that rod‐shaped NPs have better tumor‐targeting potential than spherical NPs, owing to greater cellular adhesion.^[^ ^23^, ^29^ ^]^

The second is the prolonged blood circulation time of soft HNP‐RGD. Circulation time of NPs in vivo is positively correlated with its enrichment in the tumor.^[^ ^4a^ ^]^ The relatively high blood concentration due to long blood circulation increases the probability of HNP‐RGD ending up within the tumor. In vivo circulation data showed that relative fluorescence intensity of soft HNPs was higher than hard HNPs at each time point, suggesting the longer circulation time of soft HNPs (Figure S16, Supporting Information). The longer blood circulation of soft HNPs is attributed to the soft NPs depressing clearance by the liver and physical filtration by the spleen.^[^ ^30^ ^]^ NPs with short blood circulation are quickly metabolized out of the body, so there are less residues in RES after long in vivo circulation.^[^ ^31^ ^]^ Soft HNPs were able to maintain relatively high RES residues after 24 h of in vivo circulation, also demonstrating a longer blood circulation of soft HNPs (Figure 4f). Interestingly, 15HNP‐RGD showed the longest circulation time than other HNPs (Figure S16, Table S3, Supporting Information), indicating that RGD modification can further prolong blood circulation. The increased blood circulation time of NPs by RGD modification has been previously reported, possibly because it reduces the formation of protein crowns and thus reducing NP recognition and clearance by the RES.^[^ ^32^ ^]^

It is worth noting that 15HNPs have a longer circulation time than 40HNPs, but the delivery efficiency of 15HNPs to tumor is slightly lower than that of 40HNPs (Figure 4e). A comparison of commercial PEGylated (Doxil) and non‐PEGylated liposomes (Myocet) also shows that longer blood circulation due to PEGylation does not contribute to efficacy. This is due to the fact that long‐circulating nanocarriers neither extravasate substantially to the tumor tissue nor are they cleared by the RES.^[^ ^27^ ^]^ This suggests that the active targeting design of polymeric nanocarriers may be better than passive targeting in tumor targeting therapy. Controlling the elasticity of active targeting polymeric nanocarriers can further increase their tumor active targeting potential, owing to the prolonged blood circulation and further increased cellular adhesion.

## 3 Conclusion

In summary, the elasticity of polymeric nanocarriers has a great influence on its biological properties. The in vivo experimental results demonstrated that tuning the elasticity of HNP‐RGD can improve their tumor delivery efficiency, and soft 15HNP‐RGD showed a higher targeting potential than hard 40HNP‐RGD. The enhancement of tumor targeting potential of soft polymeric nanocarriers is mainly attributed to two aspects: prolonged blood circulation and further increased cellular adhesion. Insights into the impact of elasticity on biological processes will contribute to the design and development of better polymeric nanocarriers. Polymeric nanocarriers with different elasticities will have a wider range of clinical applications.

## 4 Experimental Section

Experimental details are shown in the Supporting Information.

## Conflict of Interest

The authors declare no conflict of interest.

## Supporting information

Supporting Information

## Data Availability

The data that support the findings of this study are available in the supplementary material of this article.
