# Supplementary material for: Influence of Elasticity of Hydrogel Nanoparticles on Their Tumor Delivery
Source: Adv Sci (Weinh). 2022 Aug 18;9(29):2202644. doi: 10.1002/advs.202202644 (PMC9561785; doi:10.1002/advs.202202644)
Supplement: Supplementary file 1 — Supporting Information [file ADVS-9-2202644-s001.pdf]

ADVANCED SCIENCE
Open Access

Supporting Information

for *Adv. Sci.*, DOI 10.1002/advs.202202644

Influence of Elasticity of Hydrogel Nanoparticles on Their Tumor Delivery

Xiangyu Chen, Shuwei Zhang, Jinming Li, Xiaobin Huang, Haochen Ye, Xuezhi Qiao, Zhenjie Xue, Wensheng Yang and Tie Wang**

Supporting Information

Influence of Elasticity of Hydrogel Nanoparticles on Their Tumor Delivery

Xiangyu Chen, Shuwei Zhang, Jinming Li, Xiaobin Huang, Haochen Ye, Xuezhi Qiao, Zhenjie Xue, Wensheng Yang, Tie Wang**

Table of Contents

Materials and characterizations

Experimental methods

Supplementary figures and tables

Materials and characterizations

Materials

All chemicals were used as received without further purification. Poly(ethylene glycol) diacrylate (PEGDA, MW 600), 2-Carboxyethyl acrylate (≥ 98%), Cyclohexane (≥ 99%), Span 80, Tween 80, 2-Hydroxy-2-methylpropiophenone (≥ 97%), N,N′-Dicyclohexylcarbodiimide (DCC, ≥ 99%), N-Hydroxysuccinimide (NHS, ≥ 98%), DiI (≥ 98%), DiR (≥ 95%) and all solvent were obtained from Aladdin Ltd. (Shanghai, China). cyclo(Arg-Gly-Asp-d-Phe-Lys) (cRGDfK, ≥ 95%) were obtained from Konoscience (Beijing, China). Ultrapure water (18.2 MΩ) produced by a Millipore direct-Q system (Millipore) was used throughout the experiments.

Characterizations

The TEM images were obtained using JEOL JEM-1011 (JEOL, Tokyo, Japan) instruments at an accelerating voltage of 100 kV. The hydrodynamic sizes and zeta potentials of the HNPs were determined using a Malvern Zetasizer Nano ZS (Malvern Instruments, Malvern, UK) at a temperature of 25 °C. The Fourier transform infrared spectroscopy (FTIR) datas were obtaine using Thermo Scientific Nicolet iS20. Quantitative mechanical property mapping was carried out using a Dimension icon AFM (Bruker AXS Corporation, Santa Barbara, CA, USA), operated under the peak-force taping mode (PeakForce QNM), in which the applied force was 500 pN and the scan rate was set at 1 Hz. The spring constant of the cantilever was 0.58 N m^{-1}. The radius of the standard AFM probe was 5 nm. The spatial distribution of the Young's modulus across the nanoparticles was determined from the AFM load-displacement curves by applying the Derjaguin-Muller-Toporov (DMT) model.

Experimental method

Synthesis of hydrogel nanoparticles (HNPs)

Material was synthesized as previously reported[1]. Typically, Span 80 (300 mg) and Tween 80 (100 mg) were dissolved in cyclohexane (15 mL), and then a mixed solution of PEGDA (150 μL for the softest HNPs and 400 μL for the stiffest HNPs), water (840 μL for the softest HNPs and 590 μL for the stiffest HNPs) and 2-Carboxyethyl acrylate (10 μL) was added. The mixture was transformed to emulsion solution by ultrasonication (SCIENTZ JY92-IIN) for 1 min. Photoinitiator 2-hydroxy-2-methylpropiophenone (100 μL) was then added to the emulsion. The reaction was conducted under magnetic stirring (800 rpm) with a 365 nm longwave UV lamp for 0.5 h. The products were collected by centrifugation (12000 rpm) and washed three times with ethanol and water.

RGD Conjugation

NHS (2 mg), DCC (3 mg) and HNPs were dissolved in a mixture solvent containing $CHCl_3$ (2 mL), DMF (3 mL), and anhydrous Na_2CO_3 (10 mg). The mixture was stirred at room temperature for 30 min under argon protection. cRGDfK (2 mg in 1 mL DMF) was then added and the resulting solution was stirred overnight at room temperature under argon protection. The HNP-RGD was collected by centrifugation (12000 rpm) and washed three times with ethanol and water.

Culture of cells

Cell lines were kindly supplied by the National Center for Nanoscience and Technology in China. HUVECs (human umbilical vein endothelial cells), MCF-7 cells (human breast adenocarcinoma cells) and HeLa cells (human cervical carcinoma cells) were incubated with the DMEM medium, supplemented with 10% fetal bovine serum and 1% penicillin/streptomycin (Gibco; Thermo Fisher Scientific). RAW264.7 cells (murine macrophages) were incubated with the DMEM medium, supplemented with 10% fetal bovine serum. Cells were incubated in a cell incubator with 5% CO_2 at 37 °C.

Measuring $\alpha_v\beta_3$ integrin receptor levels on HUVECs surfaces

2×10^6 HUVECs were fixed for 1 h in 2% formaldehyde solution, then centrifuged and the supernatant was discarded. Cells were suspended in 2 mL of buffer (PBS containing 2% FBS and 0.1% sodium azide) and incubated for 30 min at 37 °C. The cell suspensions was divided equally into two eppendorff tubes, then the tubes were centrifuged and the supernatant was discarded. 100 μL of commercially available monoclonal antibodies against $\alpha_v\beta_3$ integrin receptors (at a 1:100 dilution in wash buffer) were added to one tubes and 100 μL PBS was added to another as a control. The cell suspensions were incubated at room temperature for 1

h, then centrifugated and washed with buffer. 100 μL of FITC-conjugated secondary antibody (at 1:200 dilution) was added and incubated for 45 min, then centrifugated and washed for 3 times with PBS. Finally, the HUVECs were suspended in 1 mL PBS and the flow cytometry histograms were recorded in a BD Accuri C6 Plus (BD Biosciences, San Jose, CA, USA).

Cellular uptake of HNPs

Cellular uptake of the HNPs by HUVECs, RAW264.7 cells, MCF-7 cells, and HeLa cells was studied using flow cytometry. Cells were seeded in 24-well plates (2×10^5 cells per well) and cultured for 48 h. Then, cells were coincubated with different DiI-loaded HNPs and HNP-RGD (at a concentration of around 1×10^9 particles/mL) for 1, 3, and 12 h. The cells were collected by trypsin digestion, and resuspended in 0.5 mL PBS before their fluorescence intensities were measured. Nontreated cells were regarded as the blank group. Measurement was carried out on the BD Accuri C6 Plus (BD Biosciences, San Jose, CA, USA).

For the flow experiments, cells were seeded in microfluidic devices (2×10^5 cells) and cultured for 48 h. Cells were then exposed to 300 s^{-1} shear rate in complete medium containing DiI-loaded HNPs and HNP-RGD (at a concentration of around 1×10^9 particles/mL) using a peristaltic pump. The shear rate was determined based on the flow rate and channel size using equation 1 as follow[2]:

$$\gamma = \frac{6Q}{h^2 w} \qquad \text{(equation S1)}$$

where γ (s^{-1}) is the shear rate, Q is the volumetric flow rate (0.25 mL/s), h is the channel height (0.1 cm), and w is the channel width (0.5 cm).

Cytotoxicity assays

HUVECs were seeded into a 96-well plate at a density of 1×10^5 per well and cultured for 12 h. Then the cells were incubated with HNPs at various concentrations (0, 62.5, 125, 250, 500, and 1000 μg/mL) for another 24 h. Finally, the cells were washed thoroughly with PBS and a standard CCK-8 assay was performed to measure cell viability.

***In vivo* imaging**

All animal experiments reported herein were performed under guidelines evaluated and approved by Ethics Committee of Chinese PLA General Hospital (S2020-507-02). 5-week-old female BALB/c mice were used to prepare the xenograft mouse model. 4T1 cells were collected by trypsin digestion at exponential growth phase and 1×10^7 cells in 0.1 ml of PBS were injected into the right leg region of each mouse at day 0. When the tumors grew to a size of 50-100 mm^3 (around day 10-14), mice were used for experiments. DiR-loaded 15HNPs, 15HNP-RGD, 40HNPs, and 40HNP-RGD were intravenously injected into mice through the

tail vein at a dose of 100 μL (with a DiR concentration of 100 μg/mL). At 1, 3, 6, 12, and 24 h after injection, mice were imaged using a small animal imaging system (IVIS Lumina II).

The mice were sacrificed via CO_2 overdose at 24 h and their main organs (livers, spleens, kidneys, hearts and lungs) and tumors were harvested for *in vitro* fluorescence imaging by a small animal imaging system.

***In vivo* circulation**

DiR-loaded 15HNPs, 15HNP-RGD, 40HNPs, and 40HNP-RGD were intravenously injected into mice through the tail vein at a dose of 100 μL (with a DiR concentration of 100 μg/mL). At 5 min, 30 min, 1 , 3 , 6 , 12 , and 24 h after injection, 10 μL of blood was collected through tail vein. The blood was diluted in 200 μL of heparin/PBS solution to avoid blood coagulation. The samples were centrifuged (1000 rpm) for 3 min to remove blood cells, and the supernatant was measured by a microplate reader. Two-compartment pharmacokinetics model was used to calculate in vivo circulation half-lives via PKSolver.

Statistics analysis

Results were expressed as means ± SD. Differences between groups were assessed by unpaired T test, with p values of ≥ 0.05, < 0.05, < 0.01, and < 0.001 denoted as ns, *, **, #, respectively. Statistics were calculated using GraphPad Prism 8 software.

Supplementary figures and tables

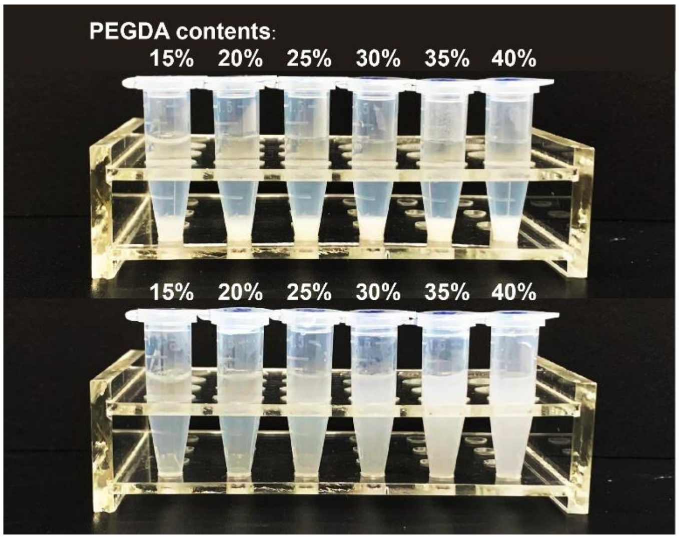

Figure S1. Full-color photograph of aqueous solutions of HNPs with different PEGDA contents. For the same number of nanoparticles, the higher the content of PEGDA, the less transmissive the solution.

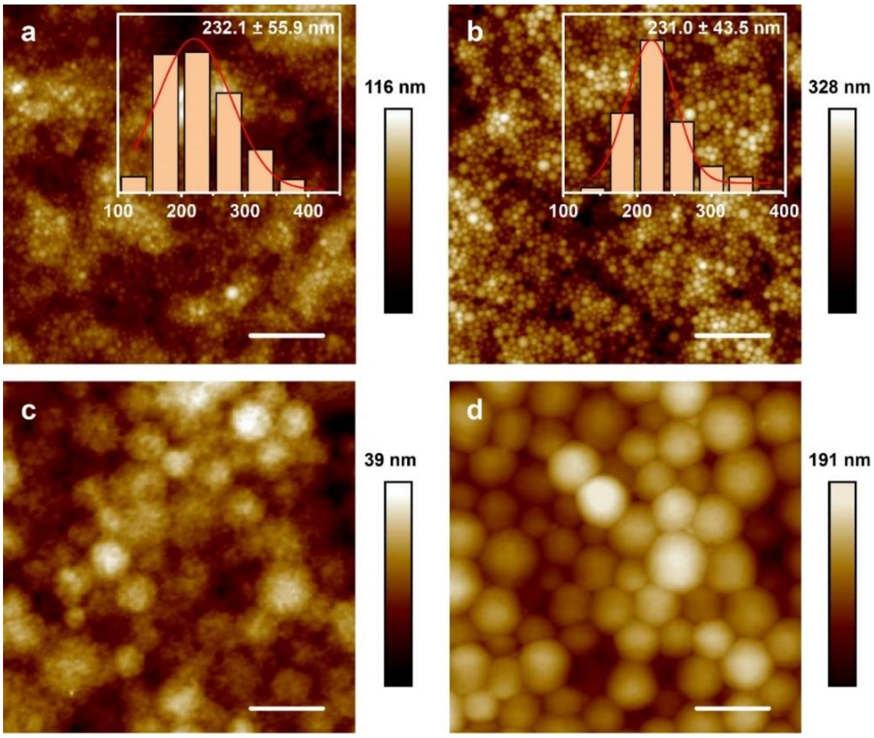

Figure S2. AFM images of (a and c) 15HNPs and (b and d) 40HNPs. The insets are size analysis of HNPs. Scale bars represent 2 μm for a and b and 400 nm for c and d.

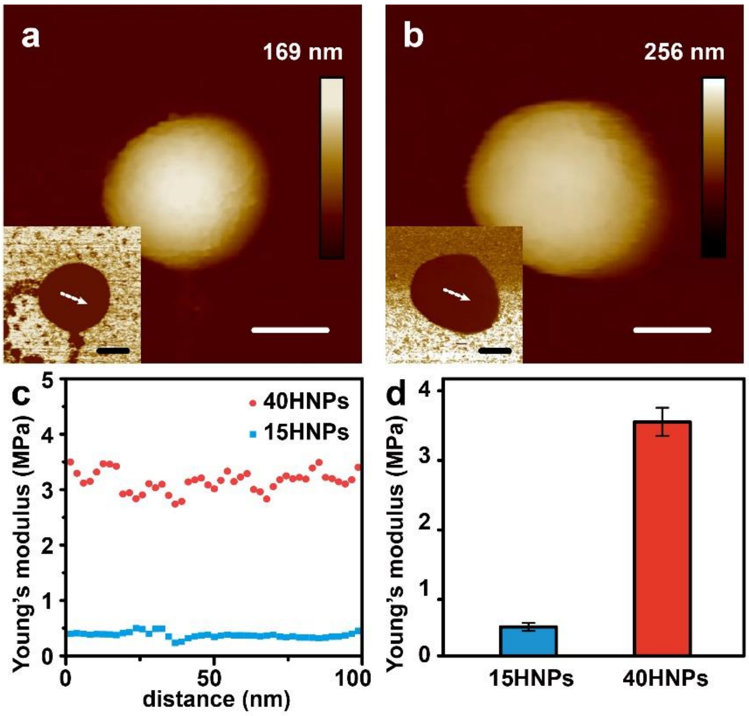

Figure S3. AFM images of (a) 15HNP and (b) 40HNP in PBS. The inset shows the corresponding Derjaguin−Mueller−Toporov (DMT) Young's modulus map. (c) Profile analysis of the Young's modulus map along the white dashed arrow in a and b. (d) The Young's modulus of HNPs. Scale bars represent 100 nm for a and b.

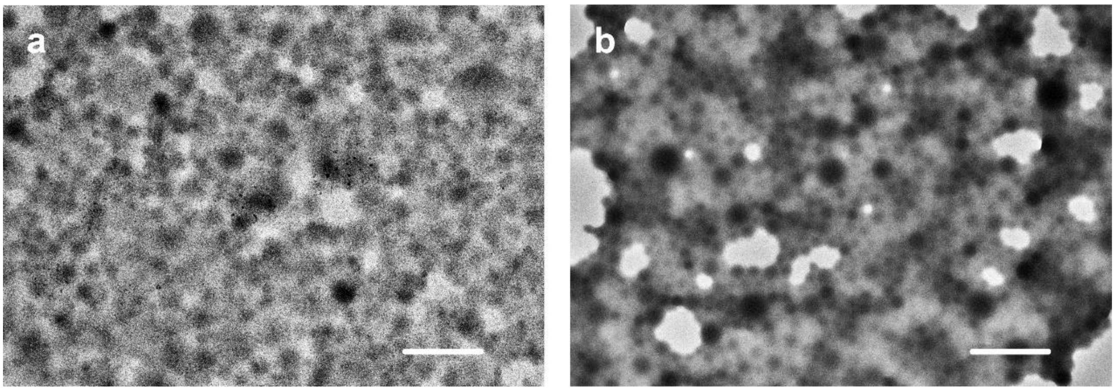

Figure S4. TEM images of (a) 15HNPs and (b) 40HNPs. 15HNPs has low contrast compared to 40HNPs because of its lower solid content. Scale bars represent 1 μm for a and b.

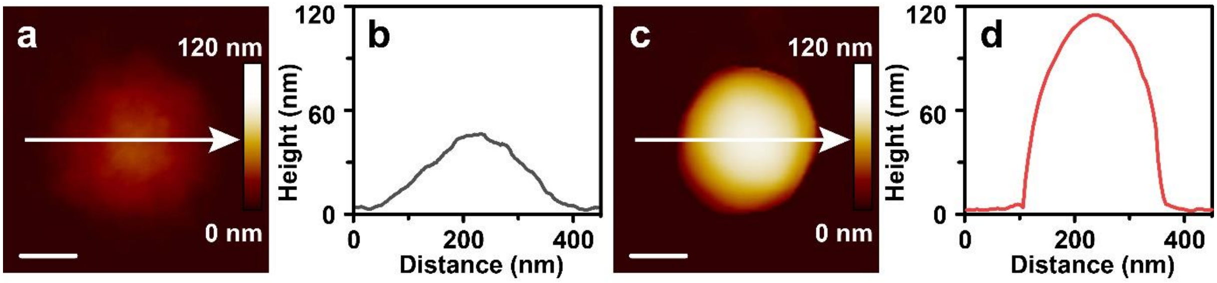

Figure S5. (a and c) AFM images of 15HNPs and 40HNPs after drying. (b and d) The cross-sectional view of the height profile corresponding to the line drawn in a and c. Scale bars 100 nm for a and c

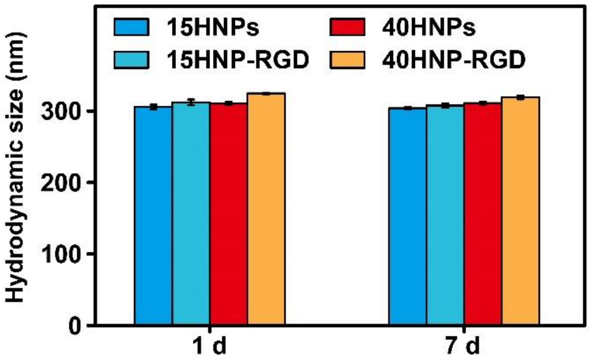

Figure S6. Stability of different HNPs in PBS solution tested by DLS. The results indicated that HNPs has good stability.

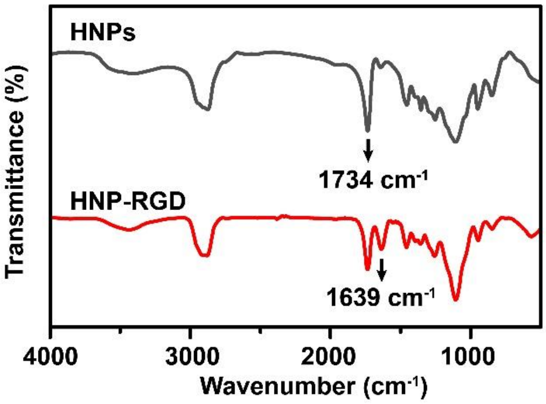

Figure S7. Fourier transform infrared spectroscopy (FTIR) examination of HNPs and HNP-RGD. The absorbance peak at peak for 1734 cm^{-1} was attributed to -C=O stretching vibration in ester group of HNPs. A characteristic peak at 1639 cm^{-1} was appeared which corresponds to the bonded amide (-CO-NH) stretching vibrations, thus confirming the successful conjugation of RGD.[3]

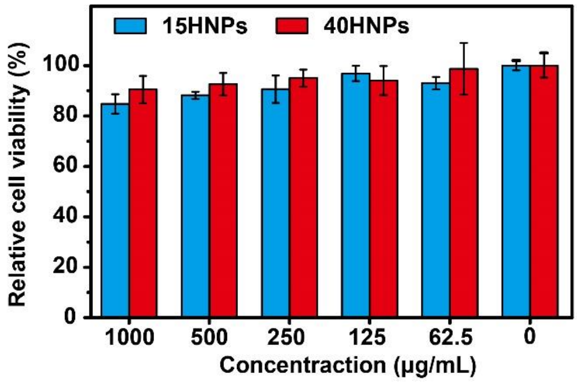

Figure S8. Cell viability analysis of HUVECs incubated with 15HNPs and 40HNPs for 24 h.

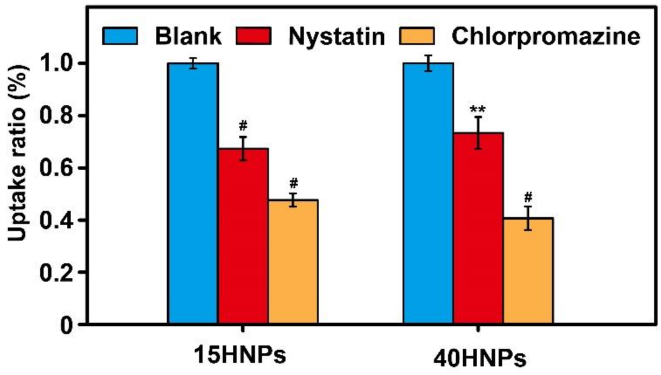

Figure S9. The investigation of cellular endocytic pathways of the HNPs using endocytosis inhibitors, including nystatin (100 U mL^{-1}) and chlorpromazine (200 mg mL^{-1}) to block their caveolin/lipid raft-mediated, and clathrin-mediated endocytosis, respectively. Values were compared to those of nontreated samples. All values are represented as the means ± SD (n = 3, with *$p < 0.05$, **$p < 0.01$, and $^{\#}p < 0.001$; ns, not significant).

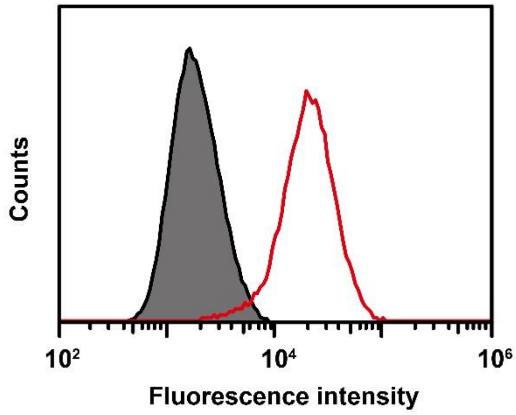

Figure S10. $\alpha_v\beta_3$ integrin receptor profiles in HUVECs measured by flow cytometry. The result showed that HUVECs displayed a great integrin $\alpha_v\beta_3$ expression.[4]

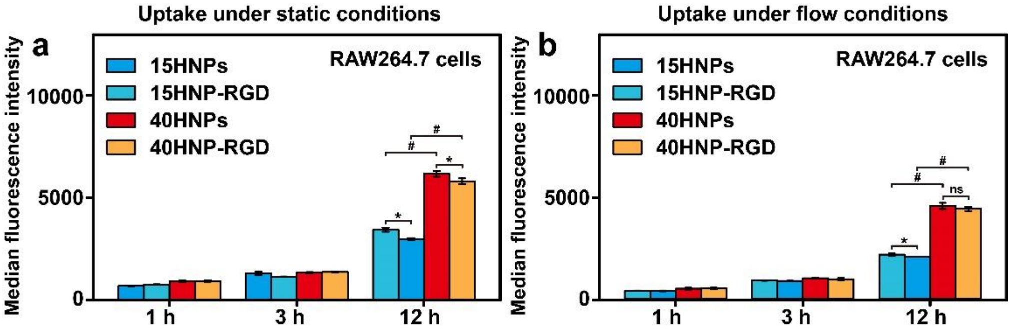

Figure S11. Cellular uptake of 15HNPs, 15HNP-RGD, 40HNPs, and 40HNP-RGD in RAW264.7 cells at various time points. All values are represented as the means ± SD (n = 3, with *p < 0.05, **p < 0.01, and $^{\#}$p < 0.001; ns, not significant).

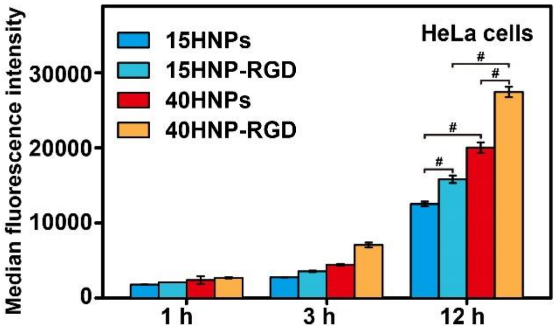

Figure S12. Cellular uptake of 15HNPs, 15HNP-RGD, 40HNPs, and 40HNP-RGD in HeLa cells at various time points. All values are represented as the means ± SD (n = 3, with *p < 0.05, **p < 0.01, and $^{\#}$p < 0.001; ns, not significant).

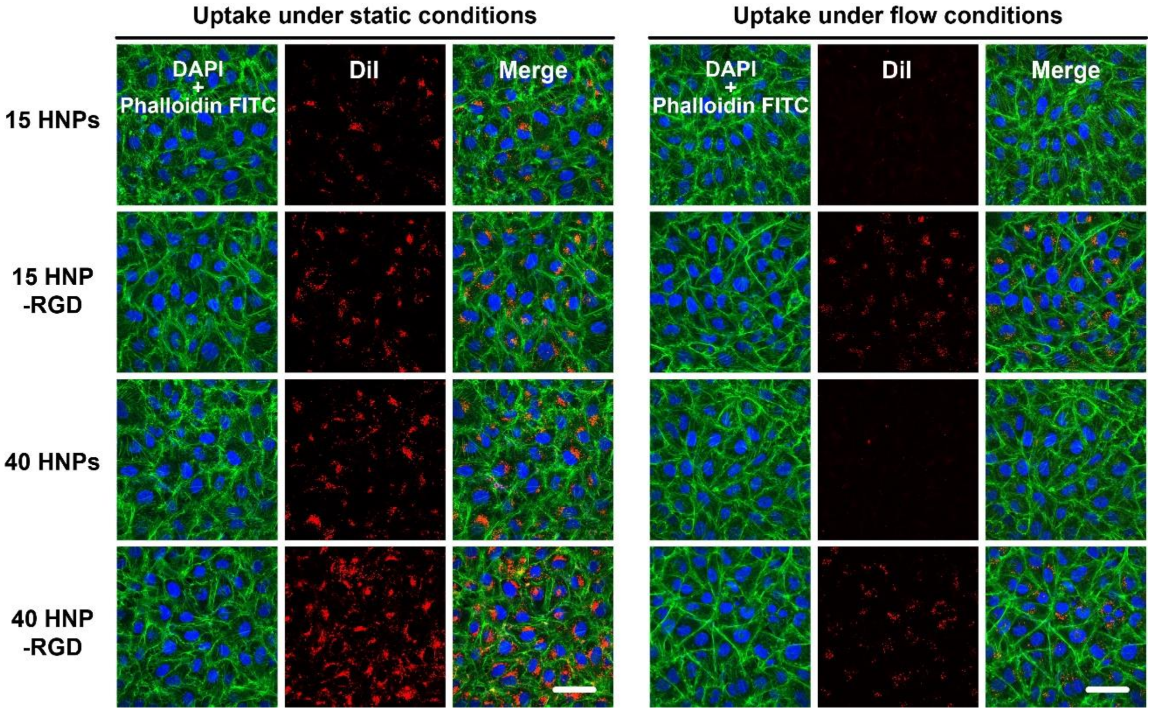

Figure S13. Fluorescence microscopic images displaying different HNPs uptake by HUVECs under static and flow conditions. Nuclei are stained blue, cytoskeleton (F-actin) is stained green and HNPs are depicted red. Scale bars represent 50 μm.

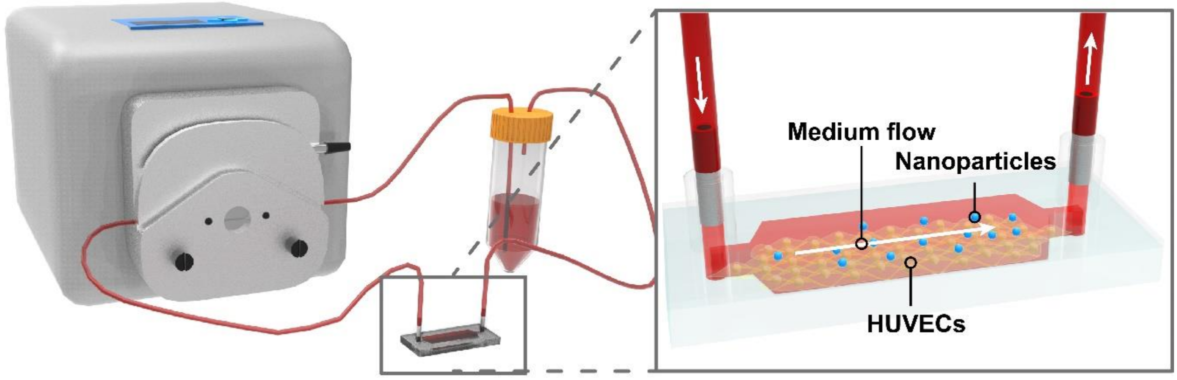

Figure S14. Schematic representation of the experimental setup for cellular uptake under flow conditions.

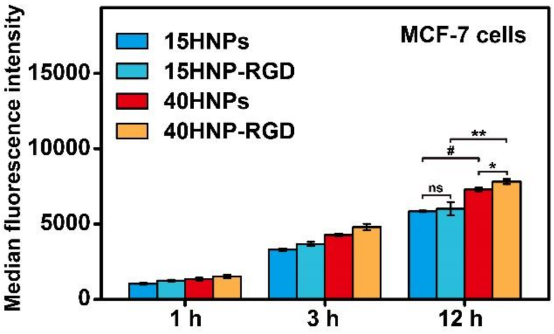

Figure S15. Cellular uptake of 15HNPs, 15HNP-RGD, 40HNPs, and 40HNP-RGD in MCF-7 cells at various time points. All values are represented as the means ± SD (n = 3, with *p < 0.05, **p < 0.01, and #p < 0.001; ns, not significant).

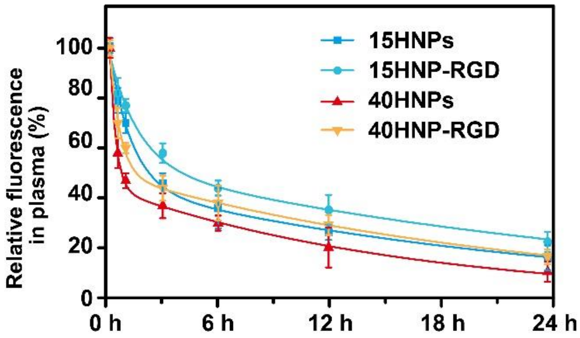

Figure S16. *In vivo* circulation of 15HNPs, 15HNP-RGD, 40HNPs, and 40HNP-RGD at different time points.

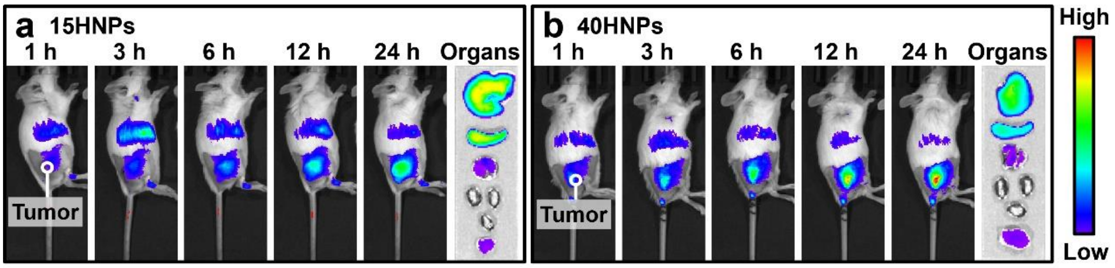

Figure S17. *In vivo* images of 4T1 tumor xenograft bearing mice at different time points and ex vivo images of various organs (liver, spleen, lung, kidneys, heart and tumor from top to bottom) collected from mice at 24 h after the injection of (a) 15HNPs and (b) 40HNPs.

Table S1. Synthesis compositions and density of different HNPs.

	15HNPs	20HNPs	25HNPs	30HNPs	35HNPs	40HNPs
%PEGDA	15	20	25	30	35	40
Density (g/cm^3)	1.018	1.024	1.030	1.036	1.042	1.048

Table S2. Size, PDI, and Zeta Potential of different HNPs

	15HNPs	15HNP-RGD	40HNPs	40HNP-RGD
Z-averaged diameter [nm]	306.1±3.1	312.4±4.1	310.9±2.1	325.0±1.1
PDI	0.10	0.09	0.11	0.14
Zeta potential [mV]	-42.7±0.1	-39.3±0.6	-40.2±0.6	-36.2±0.5

Values are means ± SD (n = 3)

Table S3. Compartmental analysis of HNPs

	15HNPs	15HNP-RGD	40HNPs	40HNP-RGD
Distribution half-life [h]	0.84	1.10	0.23	0.39
Elimination half-life [h]	16.83	20.71	11.37	15.94
MRT [h]	22.91	28.63	15.89	22.39

MRT: mean residence time.

References

[1] A. C. Anselmo, M. Zhang, S. Kumar, D. R. Vogus, S. Menegatti, M. E. Helgeson, S. Mitragotri, *Acs Nano* **2015**, *9*, 3169.

[2] W. J. Kelley, P. J. Onyskiw, C. A. Fromen, O. Eniola-Adefeso, *ACS Biomater. Sci. Eng.* **2019**, *5*, 6530.

[3] G. C. Ingavle, S. H. Gehrke, M. S. Detamore, *Biomaterials* **2014**, *35*, 3558.

[4] G. Mondal, S. Barui, A. Chaudhuri, *Biomaterials* **2013**, *34*, 6249.
